# Hydrogen-Generating Silica Material Prevents UVA-Ray-Induced Cellular Oxidative Stress, Cell Death, Collagen Loss and Melanogenesis in Human Cells and 3D Skin Equivalents

**DOI:** 10.3390/antiox10010076

**Published:** 2021-01-08

**Authors:** Li Xiao, Mai Mochizuki, Taka Nakahara, Nobuhiko Miwa

**Affiliations:** 1 Department of Pharmacology, School of Life Dentistry at Tokyo, The Nippon Dental University, Tokyo 102-8159, Japan; 2 Department of Life Science Dentistry, School of Life Dentistry at Tokyo, The Nippon Dental University, Tokyo 102-8159, Japan; mai-m@tky.ndu.ac.jp; 3 Department of Developmental and Regenerative Dentistry, School of Life Dentistry at Tokyo, The Nippon Dental University, Tokyo 102-8159, Japan; t.nakahara@tky.ndu.ac.jp; 4 Faculty of Life Sciences, Prefectural University of Hiroshima, Hiroshima 727-0023, Japan; jpn.cntr.antiaging.medsci2002@leto.eonet.ne.jp

**Keywords:** hydrogen, silica, UVA, apoptosis, necrosis, collagen, melanogenesis, pigmentation, oxidative stress

## Abstract

Ultraviolet-A (UVA) irradiation induces harmful effects on skin cells and accelerates skin aging through oxidative stress. In this study, the effects of a hydrogen-generating silica material named ULH-002 against UVA injuries in human cells and 3D skin equivalents were investigated. The oxygen radical absorption capacity (ORAC) assay showed that both freshly prepared ULH-002 solutions and 7-day-old solutions exhibited equal peroxyl radical (ROO·) scavenging activities concentration-dependently. CellROX^®^ green/orange staining showed that ULH-002 could reduce UVA-induced oxidative stress in human keratinocytes HaCaT and human gingival fibroblasts (HGFs). ULH-002 significantly prevented UVA-induced apoptotic/necrotic cell death and cell-viability decline in HGFs and keratinocytes, as shown by Annexin V/PI apoptosis assay and PrestoBlue assay, respectively. Immunostaining showed that ULH-002 prevented the UVA-induced deterioration of expression of both type IV and I collagens in the 3D skin equivalents, and similarly in monolayer HGFs. UVA-enhanced melanogenesis was observed in human melanocytes HMV-II and HMV-II cell-containing 3D skin equivalents, but markedly prevented by ULH-002 as demonstrated by Fontana–Masson’s staining. In conclusion, our data suggested that ULH-002 could protect human keratinocytes and fibroblasts from UVA-induced injuries, prevent the loss of type IV and I collagens, as well as reduce melanogenesis. ULH-002 might be developed as a skin care reagent in the cosmetic industry.

## 1. Introduction

Oxidative stress is the main contributor to skin aging processes. Environmental factors (such as UV exposure, cigarette smoke and air pollutants) induce the massive generation of reactive oxygen species (ROS) in skin cells. The overproduction of ROS overwhelms antioxidant defense systems (including enzymatic and nonenzymatic antioxidants) in the cells. As a result, oxidative stress occurs [1,2,3,4]. ROS include superoxide anion (O_2_^•−^), hydroxyl radical (^•^OH), hydrogen peroxide (H_2_O_2_), hypochlorous acid (HOCl), peroxyl radicals (ROO^•^) and hydroperoxyl radical (HOO^•^). These oxidants can damage cell components such as nucleic acids, lipids, proteins and carbohydrates, deteriorate cellular functions and finally cause cell death [5,6].

Keratinocytes, fibroblasts and melanocytes are the main cell-types in human skin. Oxidative stress-induced damage in these cells leads to cell number drop, collagen synthesis decline and pigmentation [7,8]. 

Recently, molecular hydrogen (H_2_) has gained attention as a selective antioxidant against toxic ROS. Hydrogen can react with the most toxic ROS, hydroxyl radicals (^•^OH), and detoxify them to harmless water (2 ^•^OH + H_2_ → 2H_2_O). Many studies have reported that H_2_ not only exhibits antioxidative stress effects, but also has various anti-inflammatory and anti-allergic effects [9,10,11,12]. In this study, we tested the effects of hydrogen-generating material named ULH-002 containing hydrogen-occluded microporous silica on ultraviolet-A (UVA)-induced cellular ROS generation, cell death, collagen loss and melanogenesis in human keratinocytes, fibroblasts, melanocytes and three-dimensional skin equivalents.

## 2. Materials and Methods 

### 2.1. Preparation of ULH-002 Solution

ULH-002 powder was obtained from Unilife Japan Co., Ltd. (Tokyo). The ingredients of ULH-002 are listed in Table 1. ULH-002 powder was mixed with double distilled water (DDW) (0.1 g/mL) and vibrated for 30 min. The suspension was then filtered through a 0.22 µm-pore filter. The filtered ULH-002 solution was stored at 4 °C until use. For 1-week-old ULH-002 solutions, the suspension was kept at room temperature for 1 week. 

### 2.2. Oxygen Radical Absorbance Capacity (ORAC) Assay

The oxygen radical absorbance capacity (ORAC) assay kit was purchased from Cell Biolabs Inc. USA. The reaction was performed according to the modified manufacturer’s protocol. The water-soluble vitamin E analog Trolox^TM^ was used as the antioxidant standard. Briefly, diluted ULH-002 solutions at graded concentrations (25 µL) and standards (25 µL) were added into the wells of a microplate (black 96-well plates, Nunc^TM^ black microwell, Japan). The 2,20-Azobis(2-amidinopropane) dihydrochloride (AAPH) solution (25 µL; 12 mM final concentration) and fluorescein (150 µL; 70 nM final concentration) solution were mixed immediately before the reaction and added into the wells, rapidly using a multichannel pipette. The plate was immediately placed in a fluorescence microplate reader (SH-9000Lab, HITACHI, Tokyo), and the fluorescence intensities were recorded every 5 min for 60–90 min at 37 °C with an excitation wavelength of 480 nm and an emission wavelength of 520 nm. A blank using phosphate buffer instead of the antioxidant solution and six calibration solutions using Trolox^TM^ (0, 12.5, 25, 50, 100, 200 µM) as the standard antioxidant were also carried out in the same run. All reaction mixtures were prepared in triplicate, and at least three independent runs were performed for each sample. 

### 2.3. Cell Culture

Human gingival fibroblasts (HGFs) were isolated from human gingival tissue. The tissue was obtained from 17–26-year-old patients while they received dental surgeries at the Nippon Dental University Hospital in Tokyo. Informed consent from all participants were obtained before the surgeries. The experimental processes were under approved guidelines set by the Committee of Ethics, the Nippon Dental University School of Life Dentistry in Tokyo (authorization number: NDU-T2019-01). In a manner similar to our previous reports, the gingival tissue was washed first in ice-cold PBS (-) and then ice-cold maintenance medium (MEM-α (Thermo Fisher Scientific, Tokyo) containing 20% FBS, 100 units/mL penicillin, 10 mg/mL streptomycin and 1% Gibco^®^ GlutaMAXTM Supplement (Thermo Fisher Scientific)), the tissue was minced into 1 to 3 mm^2^ fragments, plated on 10 cm dishes with the maintenance medium, and cultured at 37 °C in a humidified tissue culture incubator with 5% CO_2_. After 7–10 days of cultivation, the plastic-adherent confluent cells were treated with 0.025% trypsin containing 0.01% EDTA for 5 min to harvest pure fibroblasts (the cell number was about 1 – 5 × 10^6^ cells in one dish). HGFs were passaged and continuously sub-cultured and maintained in the maintenance medium. HGFs from the third to 14 passages were used in the experiments (Supplemental Figure S1) [13].

Human immortalized skin epidermal keratinocytes (HaCaT) were kindly provided from Professor Norbert E. Fusenig of Deutsches Krebsforschungszentrum (Heidelberg, Germany) [14]. Cells were maintained in Dulbecco’s modified Eagle’s medium (DMEM) (Gibco) supplemented with 10% FBS, 1% Gibco^®^ GlutaMAX™ Supplement (Thermo Fisher Scientific), 100 units/mL penicillin and 10 mg/mL streptomycin in a 5% CO_2_-atmosphere at 37 °C. Cells were passaged twice a week [13]. 

Human melanoma cells HMV-II were kindly gifted by Dr. Tsutomu Kasuga [15]. HMV-II cells were plated onto CellMatrix I-P (Nitta gelatin Inc., Osaka, Japan)-precoated 10 cm tissue culture dishes in Ham F12 medium (Sigma-Aldrich, Tokyo) containing 15% FBS, 100 units/mL penicillin and 10 mg/mL streptomycin in a 5% CO2-atmosphere at 37 °C. Cells were passaged twice a week [16]. 

### 2.4. Flow Cytometry 

To characterize HGFs, we assessed the expression of mesenchymal stem/stromal cell markers CD29, CD44 and CD90, hematopoietic stem cell marker CD34 and fibroblasts maker TE-7 by flow cytometry. HGFs were detached from the culture surface by 0.025% trypsin-0.01% EDTA. After being washed two times with PBS (-), the cells were incubated with primary antibodies for 60 min and secondary antibodies (for indirectly conjugated antibody) for another 30 min. The following antibodies were used for flow cytometric analysis: directly conjugated antibodies include anti-CD29-APC (clone MAR4, BD Biosciences, San Jose, CA, USA), anti-CD90-APC (clone 5E10, Sony Biotechnology Inc., Tokyo, Japan), anti-CD34-FITC (clone 581, Beckman Coulter, Fullerton, CA, USA) and anti-CD44-FITC (clone MEM-263, Thermo Scientific, Middletown, VA, USA). The indirectly conjugated antibody was anti-TE-7 (CBL271; Millipore). Alexa Fluor 647-conjugated donkey anti-mouse IgG (H + L) was used as the secondary antibody. The cells were then analyzed by a BD FACSMelody™ Cell Sorter equipped with BD FACS Chorus software (BD Bioscience). The data were further analyzed with FlowJov10.6.1 software (Tree Star, Ashland, OR, USA), as previously reported [17,18].

### 2.5. Three-Dimensional (3D) Culture of Skin Equivalents

The skin equivalents were prepared by two methods: fibrinogen gel-based method for investigating the expression of type I and IV collagens, and collagen gel-based method for the evaluation of melanogenesis.

#### 2.5.1. Fibrinogen Gel-Based Method

The fibrinogen gel-based method is similar to the one used in our previous report [13]. HGFs (1 × 10^6^ cells/mL) were gently added into 1% fibrinogen (341576, Sigma-Aldrich, Tokyo) solution. After mixing with 1 Unit/mL thrombin (605190, Sigma-Aldrich), the HGF–fibrinogen–thrombin solution was immediately added on the top of an atelocollagen sheet (Integrin^®^ sheet, KOKEN CO., LTD. Tokyo, Japan) which was placed in a 24-well culture insert with 8 μm pores (BD Falcon) in a 24-well plate. The mixture was then kept at 37 °C to form the fibrinogen gel. After 10 min, medium A (MEM-α containing 10% FBS, 250 µM 2-O-a-D-glucopyranosyl-L-ascorbic acid (AA-2G) (SMB00390, Sigma-Aldrich), 100 units/mL penicillin, 10 mg/mL streptomycin and 1% Gibco^®^ GlutaMAX^TM^ Supplement (Thermo Fisher Scientific)) was added into the culture and incubated for 4 weeks. Then, HaCaT cells (1 × 10^6^ cells/mL) were seeded on the HGFs–fibrinogen gel substrate in medium A. After being further cultivated for 3 days, the culture surfaces were exposed to air in the incubator. The cultivation was continued for another 2–4 weeks. The medium was freshly changed twice a week. 

#### 2.5.2. Collagen Gel-Based Method

The skin equivalents were prepared with a Cellmatrix type I-A culture kit (Nitta Gelatin, Japan) in a manner similar to our previous study [13]. Briefly, HGFs (1 × 10^6^ cells/mL) were gently added into the type I-A collagen gel mixed with 10% 10× concentrated MEM-alpha and 10% reconstruction buffer (2.2 g of NaHCO_3_ and 4.47 g HEPES in 100 mL 0.05 N NaOH). The mixture was seeded into a Falcon^®^ 6-well culture insert (Corning Inc., Tokyo, Japan) and placed into a 6-well plate. After the mixture was incubated in the HGFs growth medium for 3–4 days, the HaCaT cells (1 × 10^6^ cells/mL) and HMV-II (0.1 × 10^6^ cells/mL) were mixed and seeded on the HGF-collagen gel substrate and the medium was changed to KSR medium (MEM-alpha containing 15% KnockOut™ serum replacement, 100 units/mL penicillin, 10 mg/mL streptomycin and 1% Gibco^®^ GlutaMAX™ Supplement) containing 5% FBS. One day later, the medium was replaced with KSR medium containing 1% FBS. After further cultivation for 4 days, the culture surfaces were exposed to air in the incubator. The culture medium was replaced with KSR medium without FBS and incubated for another 2–4 weeks.

### 2.6. UVA Irradiation

Cells and the 3D skin equivalents were radiated with UVA by using a UVA lamp (wavelength 365 nm). The irradiation was performed 4 times at different positions of the culture well to prevent uneven irradiation. The total ultraviolet intensity was 32 J/cm^2^ or 8 J/cm^2^.

### 2.7. Cellular ROS Detection

HaCaT cells and HGFs were pre-treated with ULH-002 at different concentrations for 2 h. Then, the cells were irradiated with UVA (32 J/cm^2^). At 2 h after UVA irradiation, cellular ROS generation in cells was detected with the CellROX^®^ Orange or Green Reagent (Thermo Fisher Scientific) according to the manufacturer’s recommended protocol. ROS production in cells was observed by the EVOS^®^ FL Cell Imaging System (Thermo Fisher Scientific). To qualitatively analyze the ROS production, HaCaT cells and HGFs were detached from the culture substratum-surface by 0.025 *w/v*% trypsin–0.01% EDTA solution and then suspended in PBS (-) at a concentration of 1 × 10^6^ cells/mL. In addition, 25 µL of the cell suspension was infused into a Tali™ Cellular Analysis Slide (T10794, Thermo Fisher Scientific, Tokyo) and analyzed by the Tali™ Image Cytometer (Thermo Fisher Scientific, Tokyo, Japan). 

### 2.8. Apoptosis Detection

UVA-induced apoptosis in HGFs was detected by a Tali Apoptosis Kit - Annexin V Alexa Fluor 488 and propidium iodide (PI) (A10788, Thermo Fisher Scientific, Tokyo, Japan) according to the manufacture’s protocol. After being washed by PBS (-) twice, cells (5 × 10^5^ to 1 × 10^6^ cells/mL) were stained with Annexin V solution for 20 min in the dark. Cells were then stained with PI solution for 1–5 min. The intensity of the green and red fluorescence was measured with the Tali™ Image Cytometer (Thermo Fisher Scientific, Tokyo, Japan).

### 2.9. Cell Viability Assay

Cell viability in HGFs and HaCaT cells were measured by the PrestoBlue^®^ Assay according to the manufacturer’s protocol. At the end of the cultivation, the cells were incubated for 3 h at 37 °C with fresh medium supplemented with 10% PrestoBlue^®^ (*v/v*; A13261, Thermo Fisher Scientific). The PrestoBlue^®^ assay is based on reducing degrees of the non-toxic, cell-permeable and redox-high-sensitive dye resazurin by the intracellular sol of viable cells, which was measured by using a microplate reader (SH-9000Lab, HITACHI) with excitation/emission = 560 nm/590 nm [19] and is expressed as fluorescence intensity units.

### 2.10. Immunohistochemistry

At the end of the cultivation, HGFs and the skin equivalents were fixed in 3 mL of 4% paraformaldehyde at 4 °C overnight. The skin equivalents were then dehydrated by a series of ethanol and xylene and followed by paraffin embedding. The paraffin tissue blocks were sectioned to 5 µm-thick slices by a microtome. After deparaffinization and hydration, the sample slices were followed by immunostaining. Both samples of cells and skin equivalents were incubated with serum blocking solution for 60 min to suppress the non-specific binding of IgG, and then incubated with saturating levels of primary antibodies for 1 day at 4 °C. The primary antibodies used were anti-collagen IV (ab6586, Abcam, Tokyo) and anti-collagen I (ab34710, Abcam). After carefully washing with 1% Triton X-100 in PBS (-), the specimens were reacted with fluorochrome-conjugated secondary antibody (A11012, Thermo Fisher Scientific) diluted to 2 µg/mL in 1% Triton in PBS (-) with 1.5% normal blocking serum for 1 day at 4 °C in dark. The nuclei were stained with DAPI. Samples were imaged and analyzed with a confocal laser scanning microscopy (LSM700, Carl Zeiss Microscopy Co., Ltd., Tokyo, Japan). The fluorescence density of cells in more than 10 images from each group (negative control group, positive control group, and ULH-002-treated group) was quantified by ImageJ software [13].

### 2.11. Determination of Melanin Content 

Cellular melanin pigments in HMV-II cells were visualized with the Fontana–Masson stain. The results were observed with a light microscope. The amount of intracellular melanin granules in more than 10 images from each group was quantitatively analyzed by ImageJ software. 

### 2.12. Statistical Analysis

Statistical analysis was carried out similarly to our previous report [20]. All data, expressed as the mean ± SD, were analyzed statistically by GNU PSPP Statistical Analysis Software (version 0.8.2-gad9374) (https://www.gnu.org/software/pspp/) and EZAnalyze Excel-based tools (http://www.ezanalyze.com/). One-way analysis of variance was followed by the post hoc tests (including Tukey’s test and Bonferroni Correction). Statistical significance was considered when *p* < 0.05. All experiments were repeated 3–5 times independently with n = 5 – 10.

## 3. Results

### 3.1. ULH-002 Has Excellent Antioxidant Capacity under Cell-Free Conditions and Decreases Intracellular ROS in HaCaT Keratinocytes and HGFs

**Figure 1:**
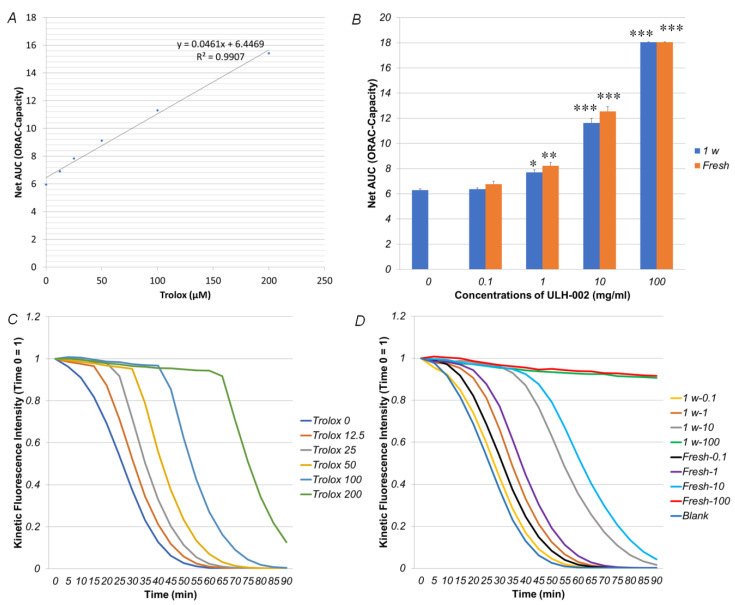
Peroxyl radical (ROO^•^) scavenging activity of ULH-002. Peroxyl radical scavenging activity of ULH-002 solutions was measured with the oxygen radical absorbance capacity (ORAC) assay. (**A**), standard curve of the ORAC assay. (**B**), ULH-002 solutions were prepared at different concentrations and left at the room temperature for one week. The control solutions were freshly prepared at the same concentrations. Both one-week-old (1 w) and fresh solutions (Fresh) were reacted with fluorescein and AAPH solutions. Histograms represent the average ORAC capacity of each solution from three independent experiments. Data were expressed as the mean ± SD. * *p* < 0.05, ** *p* < 0.01, *** *p* < 0.001 vs. “0”. (**C**), time courses of the reaction of fluorescein (FL) with AAPH in the presence of trolox at different concentrations. (**D**), time courses of the reaction of FL with AAPH in the presence of different samples. 1 w-0.1, one week old ULH-002 solution at 0.1 mg/mL; 1 w-1, one week old ULH-002 solution at 1 mg/mL; 1 w-10, one week old ULH-002 solution at 10 mg/mL; 1 w-100, one week old ULH-002 solution at 100 mg/mL; Fresh-0.1, fresh ULH-002 solution at 0.1 mg/mL; Fresh-1, fresh ULH-002 solution at 1 mg/mL; Fresh-10, fresh ULH-002 solution at 10 mg/mL; Fresh-100, fresh ULH-002 solution at 100 mg/mL.

To examine whether ULH-002 has antioxidant capacities, we first evaluated the peroxyl radical (ROO^•^) scavenging activity (ORAC capacity) in ULH-002 under cell-free conditions. As shown in Figure 1A,C, the standard curve and kinetic fluorescence intensity showed a good correlation between Net-AUC (ORAC capacity) and Trolox concentrations. Figure 1B,D showed that the ORAC capacity of ULH-002 solutions was increased in a concentration-dependent manner. In our previous study, we demonstrated that dissolved hydrogen diffuses out of the solution in a short time (t1/2 is about 30–60 min) [21]. Therefore, we compared the ORAC capacity in both fresh and 1-week-old ULH-002 solution. Interestingly, the 1-week-old ULH-002 solutions and fresh solutions exhibited almost the same ORAC capacity (*p* > 0.05). This result suggests that the antioxidant capacity did not decrease during storage.

**Figure 2:**
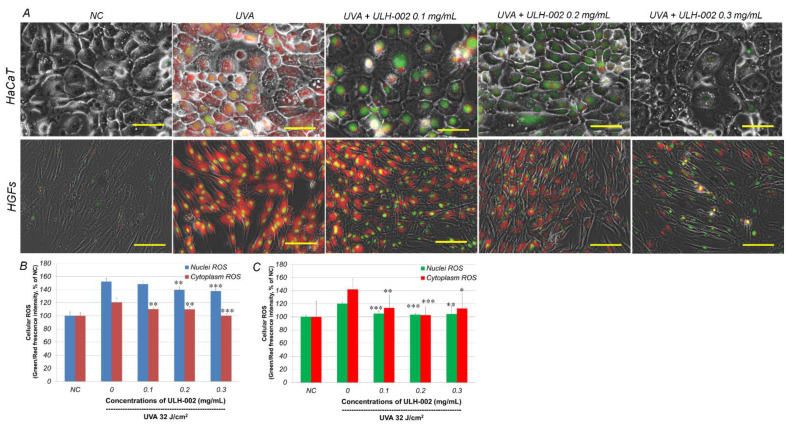
Repressive effects of ULH-002 on hydrogen peroxide-induced cellular reactive oxygen species (ROS) in human keratinocytes HaCaT and human gingival fibroblasts (HGFs). HaCaT cells and HGFs were pretreated with ULH-002 at different concentrations for 2 h. Cells were then irradiated by ultraviolet-A (UVA) at 32 J/cm^2^. At 2 h after UVA irradiation, cellular ROS was detected with CellROX^®^ green and orange dyes and observed by fluorescence microscopy. To qualitatively analyze the cellular ROS, HaCaT cells and HGFs were detached from the culture substratum-surface by trypsin and followed with Tali-cytometric analysis as described in “Materials and Methods”. (**A**), typical images of cellular ROS in HaCaT cells (upper panel) and HGFs (lower panel). Green, nuclei ROS; red, cytoplasm ROS. Scale bar indicates 10 µm in the upper panel and 25 µm in the lower panel. (**B**), Tali-cytometric analysis for HaCaT cells. (**C**), Tali-cytometric analysis for HGFs. Data were expressed as the mean ± SD. *, *p* < 0.05, **, *p* < 0.01, ***, *p* < 0.01 vs. “0”.

Then, we pretreated human keratinocytes HaCaTs and HGFs with ULH-002 at 0.1–0.3 mg/mL and irradiated the cells by UVA. Before the experiments, we first examined the cytotoxicity of ULH-002 and found out that ULH-002 did not have significant cytotoxicity under the concentration of 0.5 mg/mL, whereas ULH-002 1.0 mg/mL significantly decreased the cell viability to 82.8% of that in the negative control (*p* < 0.01) (data not shown). Therefore, we chose 0.1–0.3 mg/mL to perform the experiments of cytoprotection against UVA-injury. As shown in Figure 2, at 2 h after UVA irradiation, the intracellular ROS were increased in both cytoplasm and nuclei in HaCaT cells and HGFs. ULH-002-pretreatment significantly reduced the intracellular ROS in both HaCaT cells and HGFs. These results suggest that ULH-002 exhibited excellent antioxidant ability. 

### 3.2. Characterization of Human Gingival Fibroblasts

**Figure 3:**
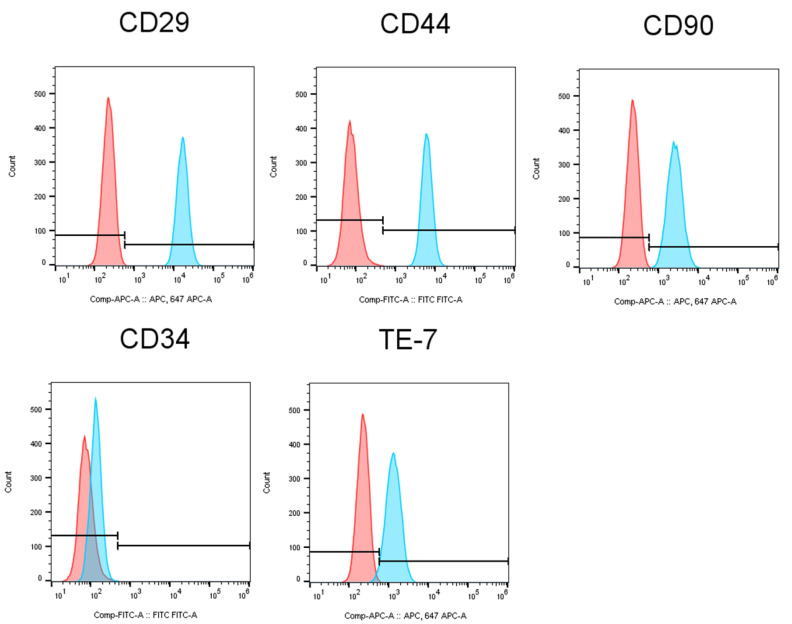
Characterization of HGFs. The expression of stem cell markers (CD29, CD44 and CD90), hematopoietic stem cell marker (CD34) and fibroblasts marker (TE-7) on the surface of HGFs was analyzed by a BD FACSMelody™ Cell Sorter. The pink histograms indicate negative control, and the blue histograms show the stained cells, respectively.

Human gingival fibroblasts are the major cells derived from gingival tissue and share many similarities with fibroblasts from other tissues, such as human dermis [22]. We characterized HGFs by using flow cytometric analysis. As shown in Figure 3, HGFs expressed mesenchymal stem/stromal markers CD29, CD90 and CD44, and fibroblasts marker TE-7. Meanwhile, hematopoietic stem cell marker CD34 was absent on the cell surface of HGFs. These data suggested that HGFs have mesenchymal stemness and a fibroblastic character which can be used for reconstructing skin tissue. 

### 3.3. Effects of ULH-002 on UVA-Induced Cell Death in HGFs and HaCaT Cells

**Figure 4:**
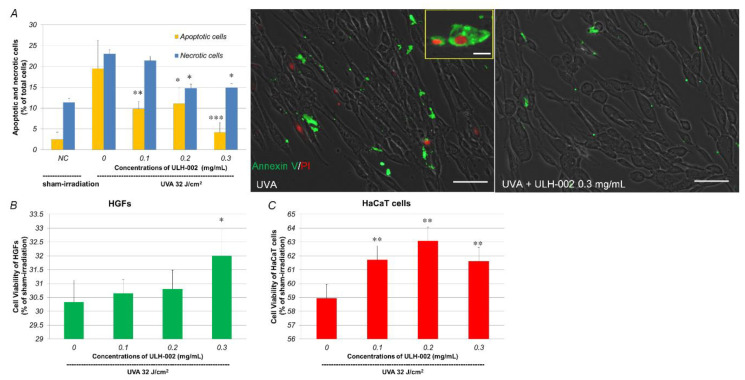
Repressive effects of ULH-002 on UVA-induced cell damage in HGFs and HaCaT cells. (**A**,**B**), HGFs were pretreated with ULH-002 at different concentrations for 2 h. Cells were then irradiated with UVA (32 J/cm^2^). At 24 h after UVA irradiation, apoptotic/necrotic cells were detected by the Tali apoptosis kit. For the Tali analysis, cells were trypsinized and followed by Annexin V/PI staining. Cells expressing green fluorescence were counted as apoptosis. Cells expressed both green and red, or red only were counted as necrosis. For images, cells were washed twice with PBS(-) and then stained with Annexin V /PI. Green, Annexin V; red, PI. Scale bar indicates 25 µm in the broad images and 10 µm in the corner image (represents necrotic cells). NC, negative control group (sham irradiation). (**B**), at 48 h after UVA irradiation, cell viability of HGFs was measured by PrestoBlue assay. (**C**), HaCaT cells were pretreated with ULH-002 for 2 h and then irradiated with UVA. At 24 h after UVA irradiation, cell viability was measured by PrestoBlue assay. Data were expressed as mean ± SD. *, *p* < 0.05, **, *p* < 0.01, ***, *p* < 0.001 vs. “0”.

UVA can reach the dermis of the skin, cause the death of dermal fibroblasts and decrease collagen synthesis [23,24]. Although keratinocytes are more resistant to UVA radiation than dermal fibroblasts, UVA also damages keratinocytes through oxidative stress [25]. We then tested the effects of ULH-002 on UVA-induced cell death in both HGFs and HaCaT cells. As shown in Figure 4A, UVA irradiation induced both apoptosis and necrosis in HGFs. There were 19.5% apoptotic cells and 23.1% necrotic cells in UVA-irradiated HGFs at 24 h post-UVA irradiation. ULH-002 (0.1–0.3 mg/mL) pretreatment significantly reduced the numbers of both apoptotic and necrotic cells, especially at 0.3 mg/mL.

UVA irradiation greatly decreased the cell viability to 30.3% of that in sham-irradiated HGFs (NC) at 48 h post-UVA irradiation (Figure 4B). UVA also markedly reduced cell viability to 58.9% in HaCaT cells (Figure 4C). ULH-002 0.1–0.3 mg/mL pre-treatment showed significant cytoprotection against UVA-induced cell death in both HGFs and HaCaT cells. ULH 0.3 mg/mL significantly increased the cell viability in both HGFs and HaCaT cells. Thus, we chose 0.3 mg/mL for the rest of the experiments. 

### 3.4. Effects of ULH-002 on UVA-Induced Collagen Synthesis Decline in HGFs

**Figure 5:**
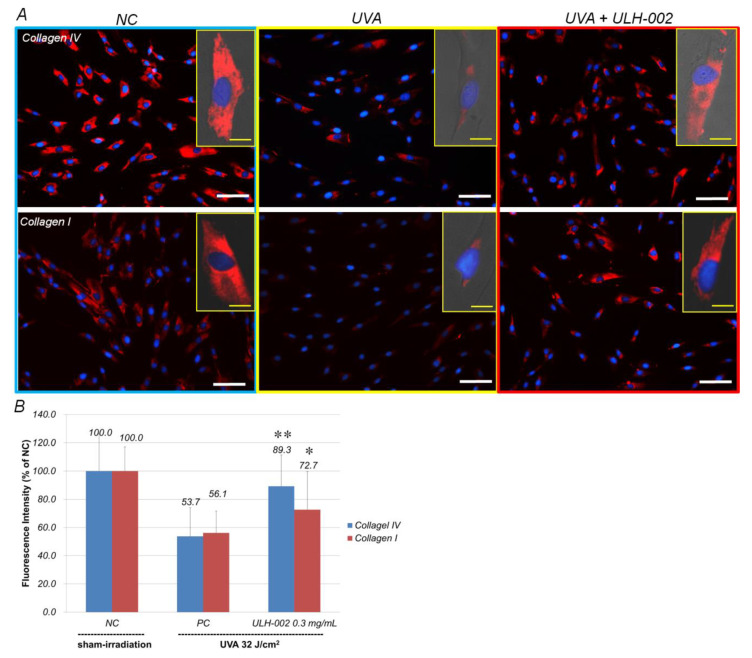
Repressive effects of ULH-002 on UVA-induced collagen loss in HGFs. HGFs were pretreated with ULH-002 at 0.3 mg/mL for 2 h and then irradiated with UVA. At 24 h after UVA irradiation, the expression of type IV and I collagens was observed by immunostaining. (**A**), images of immunostaining; blue, nuclei; red, type IV or I collagen. Images in the right top corner present single HGFs. Scale bar indicates 50 µm in the broad images and 5 µm in the corner images. (**B**), the immunostaining results were analyzed with an ImageJ software. NC, negative control group (sham irradiation); PC, positive control group. Data were expressed as the mean ± SD. *, *p* < 0.05, **, *p* < 0.01 vs. PC.

The synthesis of type IV and I collagens is a basic function of human fibroblasts [26,27]. Figure 5A showed that HGFs strongly expressed both type IV and I collagens (sham irradiation). UVA irradiation markedly decreased the expression of type IV and I collagens in HGFs to 53.7 and 56.1% of that in sham-irradiated cells, respectively. When the cells were pre-treated with ULH-002 of 0.3 mg/mL, the expression of type IV and I collagens was increased to 89.3 and 72.7%, respectively (Figure 5B). These data suggest that ULH-002 could prevent UVA injuries in the fibroblasts.

### 3.5. Effects of ULH-002 on UVA-Induced Collagen Synthesis Decline in 3D Skin Equivalents

**Figure 6:**
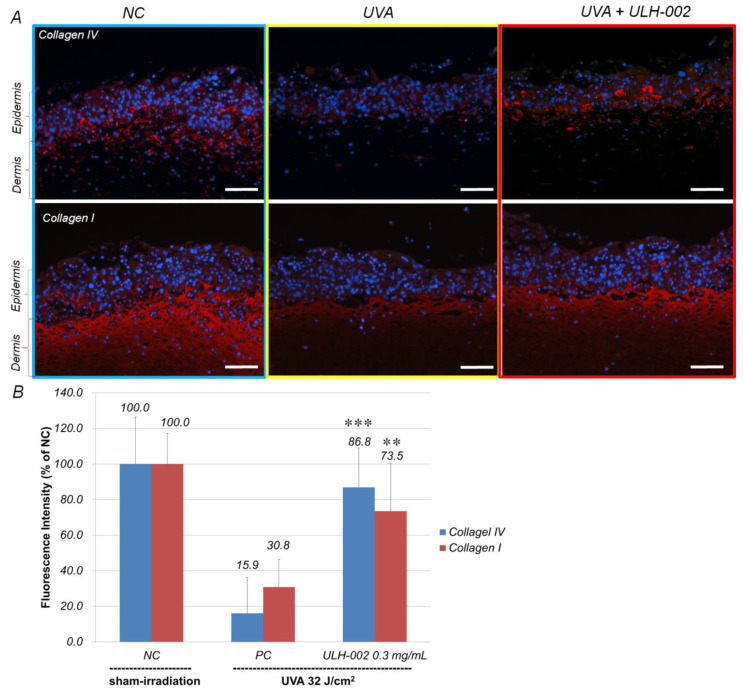
Repressive effects of ULH-002 on UVA-induced cell death and collagen synthesis decline in three-dimensional skin equivalents. The 3D skin equivalents were constructed as described in the ‘‘Materials and Methods’’. (**A**), The 3D skin equivalents were pretreated with ULH-002 at 0.3 mg/mL for 24 h. The equivalents were then irradiated with UVA (32 J/cm^2^). At 24 h after UVA irradiation, the expression of type IV and I collagens was observed by immunostaining. (**B**), The immunostaining results were analyzed with an ImageJ software. NC, negative control group (sham irradiation); PC, positive control group. Data were expressed as mean ± SD. **, *p* < 0.01, ***, *p* < 0.001 vs. PC; blue, nuclei; red, type IV or I collagen. Scale bar = 100 µm.

We then measured the effects of ULH-002 on UVA-induced collagen synthesis decline in 3D skin equivalents. Type IV collagen is known as the major structural protein of basement membranes in the skin [28]. Type I collagen is abundantly presented in the dermis. As shown in Figure 6A, by using fibrinogen gel-based method, the 3D skin expressed type IV collagen in the epidermis-dermis border loci (the location of basement membrane) and type I collagen in the dermis suggesting that the 3D skin has a similar distribution of structural proteins with human skin. UVA irradiation decreased the expression of both type IV and I collagens in the 3D skin to 15.9 and 30.8% of that in the sham-irradiated 3D skin. However, ULH-003 (0.3 mg/mL) pretreatment significantly increased the expression of type IV and I collagens to 86.8 and 73.5%, respectively (Figure 6B). 

### 3.6. Inhibitory Effect of ULH-002 on Melanin Synthesis in HMV-II Melanocytes and 3D Skin Equivalents

**Figure 7:**
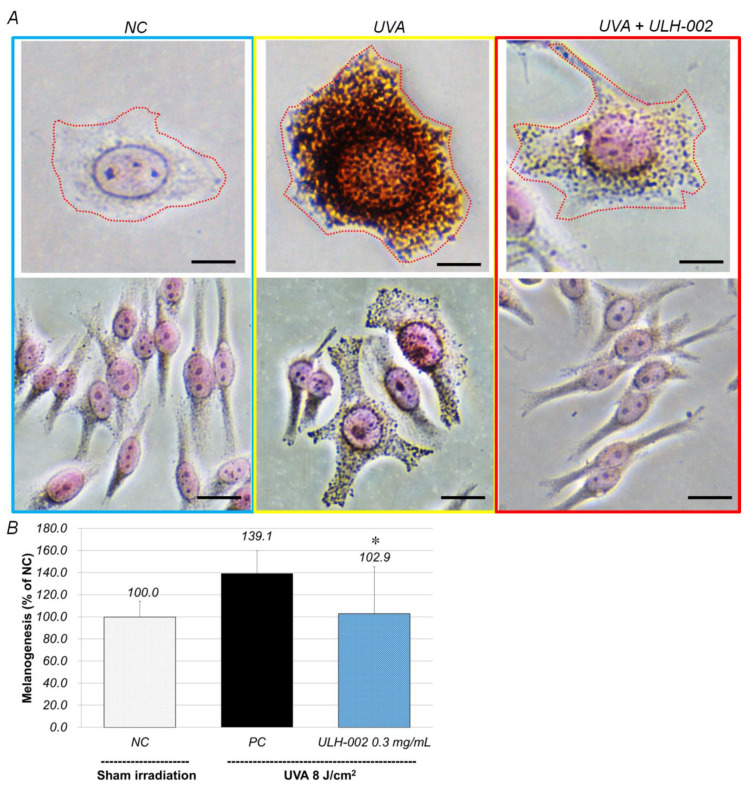
Repressive effects of ULH-002 on UVA-induced melanogenesis in HMV-II human melanocytes. (**A**), HMV-II cells were pretreated with ULH-002 at 0.3 mg/mL for 2 h. The equivalents were then irradiated with UVA (8 J/cm^2^). At 24 h after UVA irradiation, the cells were fixed by 10% formalin. Cellular melanin was stained by Fontana–Masson’s method as described in the ‘‘Materials and Methods’’. Pink, nuclei; dark brown, melanin granules; scale bar indicates 5 µm in the upper panel and 10 µm in the lower panel. (**B**), The Fontana–Masson staining results were analyzed with an ImageJ software. NC, negative control group (sham irradiation); PC, positive control group. Data were expressed as the mean ± SD. *, *p* < 0.05, vs. PC.

We examined the inhibitory effects of ULH-002 on melanogenesis in human melanoma HMV-II cells. As shown in Figure 7, at 24 h after irradiation with UVA (0.8 J/cm^2^), the melanin content in HMV-II cells was increased to 139.1% of that in the sham-irradiated cells (NC). In contrast, ULH-002 significantly reduced UVA stimulated melanin production to 102.9%. 

**Figure 8:**
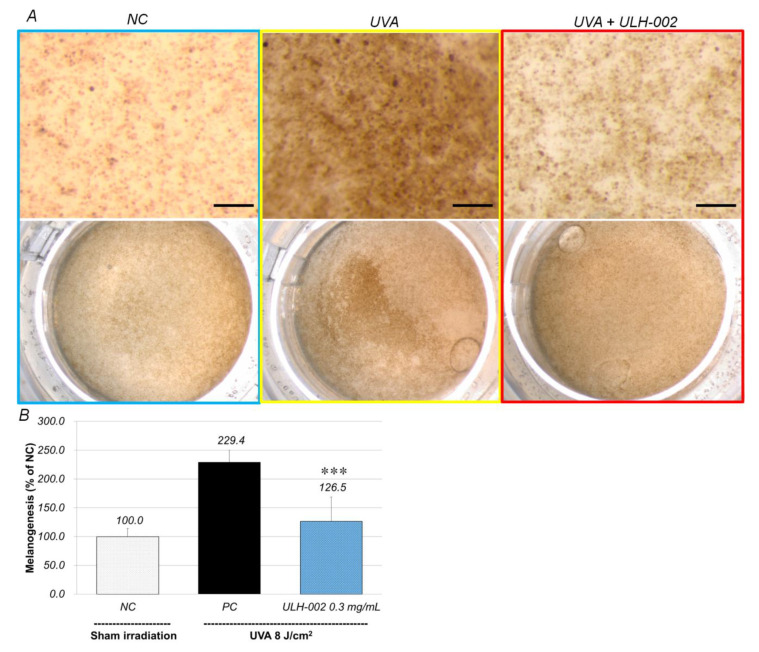
Repressive effects of ULH-002 on UVA-induced melanogenesis in three-dimensional human skin equivalents. The 3D skin equivalents were constructed as described in the ‘‘Materials and Methods’’. (**A**), the 3D skin equivalents were pre-treated with ULH-002 at 0.3 mg/mL for 24 h. The equivalents were then irradiated with UVA (8 J/cm^2^). At 24 h after UVA irradiation, the surface of 3D skin was observed by a stereoscopic microscope. The brown color indicates melanin pigments. Scale bar = 500 µm. (**B**), the pigments were analyzed with ImageJ software. NC, negative control group (sham irradiation), PC, positive control group. Data were expressed as the mean ± SD. ***, *p* < 0.001 vs. PC.

We further examined the anti-pigmentation effects of ULH-002 in the 3D skin equivalents. Under the conditions as we described in the Materials and Methods, after 3–4 weeks, HGF, HMV-II and HaCaT cells formed 3D skin equivalents with melanin pigments. We treated the skin with ULH-002 and irradiated it with UVA. As shown in Figure 8, without UVA irradiation the skin exhibited a light brown color. With UVA irradiation, the 3D skin exhibited a much darker brown color, suggesting that UVA induced melanin production in HMV-II cells in the skin. Using the ImageJ software, we quantitatively analyzed the anti-pigmentation effects of ULH-002. Figure 8B shows that as compared to the negative control, the pigmentation increased to 229.4% in UVA-irradiated skin. In contrast, with ULH-002 treatment (0.3 mg/mL), the skin color was much lighter (Figure 8A). The pigmentation in ULH-002-treated skin was reduced to 126.5% (Figure 8B).

## 4. Discussion

UV contains UVA (320–400 nm), UVB (280–320 nm) and UVC (200–280 nm). As the most harmful UV, UVC is absolved by the ozone layer. Therefore, UVA and UVB are the main contributors to skin aging. Unlike UVB (which damages cells in the skin mostly by its direct radiant effect on DNA), the less energetic UVA photons exert their harmful effects on cellular components through the generation of oxidative stress [29,30]. 

As the smallest molecule, H_2_ can easily permeate cellular membranes and selectively react with toxic ROS to reduce oxidative stress through its regulation of Ca^2+^-dependent gene expression [31]. Currently, the administration of H_2_ is mainly through drinking hydrogen-dissolved water, taking hydrogen baths or inhaling hydrogen gas. The administrated hydrogen molecules are usually rapidly eliminated through the respiratory system within few hours [32,33,34]. However, UVA-induced oxidative stress constantly occurs in the skin during the daytime, especially in summer. Thus, for cosmetic use, hydrogen needs to be stored in a media for a long period. Silica is a benefit mineral that has been widely used in cosmetic materials and food additives. Silica in micro-porous cavities of amorphous forms also is known to have hydrogen-confining storage capacity after the treatment of hot-melting and the subsequent gradual cooling under high pressure of hydrogen gas. In this study, we first tested the antioxidant capacity of silica-occluded hydrogen ULH-002 by ORAC assay. The data showed that ULH-002 solutions have an excellent ORAC capacity in a concentration-dependent manner. The ORAC capacity of ULH-002 aqueous solution was kept at the same level even after 1 week of storage at room temperature. These data suggest that silica-occluded hydrogen is very stable during a long period. In fact, ULH-002 dry powder can be stored at room temperature for years without losing its ORAC capacity (data not shown). Our previous study demonstrated that autoclaved (120 °C, 10 min) ULH-002 power or aqueous solution (the silica-occluded hydrogen gas was eliminated into the atmosphere by autoclaving, even saving other remaining ingredients) and did not show antioxidant capacity and cell-death-preventing ability [35]. We then tested if ULH-002 solutions can scavenge cellular ROS in human keratinocytes and fibroblasts. As shown in Figure 2, ULH-002 solutions (0.1–0.3 mg/mL) significantly reduced both UVA-induced cytoplasm and nuclei ROS in both HaCaT cells and HGFs, suggesting that ULH-002 can prevent oxidative stress in skin cells probably through the dual permeations into the cell membrane and nucleus membrane, during the elimination of hydrogen bubbles from the occluded silica micro-porous cavities. 

As compared to human dermal fibroblasts, human gingival fibroblasts exhibited a higher ability for wound healing [36,37]. In this study, our data showed that HGFs expressed dermal fibroblastic marker TE-7, and stem cells markers CD29, CD44 and CD90 (Figure 3). HGFs also expressed abundant type IV and I collagens (Figure 5). These data suggested that HGFs do not only present the properties of dermal fibroblasts but also wear some stem cell characters. They may have the ability to differentiate into other skin cell types when being built in skin equivalents. The harmful effects of oxidative stress on HGFs might be a reflection of other types of stem cells. Our data demonstrated that UVA at 32 J/cm^2^ (equals exposure to solar light about 5–6 h during the summertime in Tokyo) severely damaged HGFs, HaCaT keratinocytes and the 3D skin equivalents indicated by drastically reduced cell viability in HGFs and HaCaT cells, and the suppression of collagen expression in both HGFs and the skin equivalents. ULH-002 solution significantly protected HGFs, HaCaT cells and the skin equivalents from the aforementioned harmful effects of UVA. The mechanism is likely due to its antioxidative ability against UVA-induced oxidative stress. 

Melanogenesis (melanin synthesis) is one of protective responses when melanocytes are stimulated by UV radiation [38]. Epidermal melanocytes are known as instigators and victims of UV-induced oxidative stress [8]. We confirmed that weaker UVA (8 J/cm^2^) could induce melanogenesis whereas stronger UVA radiation killed melanocytes (data not shown). Figure 7 and Figure 8 showed that UVA (8 J/cm^2^) significantly increased melanogenesis in both monolayer HMV-II cells and the 3D skin equivalents, suggesting these melanocytes were under oxidative stress. When HMV-II cells and the 3D skin were pretreated with ULH-002, melanogenesis was significantly inhibited. 

## 5. Conclusions

In conclusion, ULH-002 exhibits excellent antioxidative properties, and can protect skin cells such as fibroblasts, keratinocytes and melanocytes from UVA injuries. ULH-002 is stable for a long period, and is suitable for cosmetic use, skin protection and antiaging.

## Figures and Tables

**Table 1:** Ingredients of ULH-002.

Material Composition	Amount	Origin	Gene Recombination	Allergen	BSE
Seaweed-derived calcium	0.58%	France	N/A ^1^	N/A	N/A
Perilla oil	0.20%	Japan	N/A	N/A	N/A
Potassium carbonate	42.00%	Japan	N/A	N/A	N/A
Sodium bicarbonate	33.00%	Japan	N/A	N/A	N/A
Potassium citrate	21.20%	Japan	N/A	N/A	N/A
Fine-grained silicon dioxide	Less than 3%	Korea	N/A	N/A	N/A
Magnesium sulfate	0.02%	Korea	N/A	N/A	N/A
Hydrogen-occluded microporous silica	Over 0.01%	Japan	N/A	N/A	N/A

^1^ N/A: not applicable.

## Data Availability

The data presented in this study are available within the article and its Supplementary Materials. Other data related to this study are available on request from the corresponding author.

## Supplementary Materials

The following are available online at https://www.mdpi.com/2076-3921/10/1/76/s1, Figure S1: Human gingival tissue was minced to small fragments and then cultivated with the maintenance medium for 7–10 days as descripted in materials and methods. A–C, HGFs were migrated from the fragments and proliferated after 7–10 days cultivation. Scale bars indicate 100 µm. D, Cultivated HGFs at passage 12. Scale bar indicates 25 µm.
