# Supplementary material for: Hydrogen-Generating Silica Material Prevents UVA-Ray-Induced Cellular Oxidative Stress, Cell Death, Collagen Loss and Melanogenesis in Human Cells and 3D Skin Equivalents
Source: Antioxidants (Basel). 2021 Jan 8;10(1):76. doi: 10.3390/antiox10010076 (PMC7827282; doi:10.3390/antiox10010076)
Supplement: Supplementary file 1 [file antioxidants-10-00076-s001.pdf]

Article

Hydrogen-generating silica material prevents UVA-ray-induced cellular oxidative stress, cell death, collagen loss and melanogenesis in human cells and 3D-skin equivalents

Supplemental data

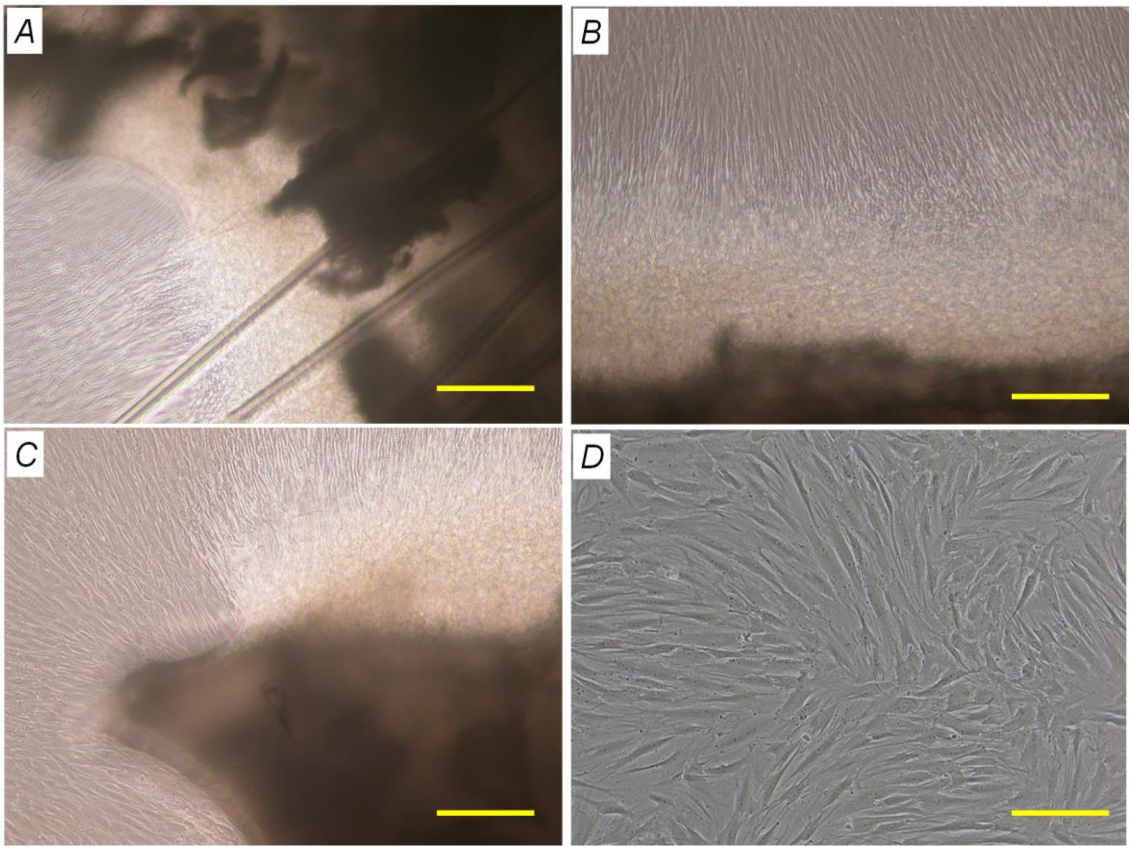

Supplemental Figure 1. Primarily cultivated HGFs

Human gingival tissue was minced to small fragments and then cultivated with the maintenance medium for 7-10 days as descripted in materials and methods. A – C, HGFs were migrated from the fragments and proliferated after 7-10 days cultivation. Scale bars indicate 100 µm. D, Cultivated HGFs at passage 12. Scale bar indicates 25 µm.
